# Circulating Levels of Fatty Acid-Binding Protein Family and Metabolic Phenotype in the General Population

**DOI:** 10.1371/journal.pone.0081318

**Published:** 2013-11-20

**Authors:** Shutaro Ishimura, Masato Furuhashi, Yuki Watanabe, Kyoko Hoshina, Takahiro Fuseya, Tomohiro Mita, Yusuke Okazaki, Masayuki Koyama, Marenao Tanaka, Hiroshi Akasaka, Hirofumi Ohnishi, Hideaki Yoshida, Shigeyuki Saitoh, Tetsuji Miura

**Affiliations:** Department of Cardiovascular, Renal and Metabolic Medicine, Sapporo Medical University School of Medicine, Sapporo, Japan; Max Delbrueck Center for Molecular Medicine, Germany

## Abstract

**Objective:**

Fatty acid-binding proteins (FABPs) are a family of 14-15-kDa proteins, and some FABPs have been to be used as biomarkers of tissue injury by leak from cells. However, recent studies have shown that FABPs can be secreted from cells into circulation. Here we examined determinants and roles of circulating FABPs in a general population.

**Methods:**

From the database of the Tanno-Sobetsu Study, a study with a population-based cohort design, data in 2011 for 296 subjects on no medication were retrieved, and FABP1∼5 in their serum samples were assayed.

**Results:**

Level of FABP4, but not the other isoforms, showed a gender difference, being higher in females than in males. Levels of all FABPs were negatively correlated with estimated glomerular filtration rate (eGFR), but a distinct pattern of correlation with other clinical parameters was observed for each FABP isoform; significant correlates were alanine aminotransferase (ALT), blood pressure (BP), and brain natriuretic peptide (BNP) for FABP1, none besides eGFR for FABP2, age, BP, and BNP for FABP3, age, waist circumference (WC), BP, BNP, lipid variables, high-sensitivity C-reactive protein (hsCRP), and HOMA-R for FABP4, and age, WC, BP, ALT, BNP, and HOMA-R for FABP5. FABP4 is the most strongly related to metabolic markers among FABPs. In a multivariate regression analysis, FABP4 level was an independent predictor of HOMA-R after adjustment of age, gender, WC, BP, HDL cholesterol, and hsCRP.

**Conclusions:**

Each FABP isoform level showed a distinct pattern of correlation with clinical parameters, although levels of all FABPs were negatively determined by renal function. Circulating FABP4 appears to be a useful biomarker for detecting pre-clinical stage of metabolic syndrome, especially insulin resistance, in the general population.

## Introduction

Intracellular lipid chaperones known as fatty acid-binding proteins (FABPs) are a group of molecules that coordinate lipid responses in cells. FABPs are abundantly expressed 14-15-kDa proteins that can reversibly bind hydrophobic ligands such as saturated and unsaturated long chain fatty acids with high affinity [1], [2]. FABPs have been proposed to facilitate the transport of lipids to specific compartments in the cell. At least nine distinct types of FABP have been identified, and each type has a characteristic pattern of tissue distribution. The FABP types are named after the tissues in which they were first identified, and the FABP family consists of liver-type (FABP1/L-FABP), intestinal-type (FABP2/I-FABP), heart-type (FABP3/H-FABP), adipocyte-type (FABP4/A-FABP), epidermal-type (FABP5/E-FABP), ileal-type (FABP6/Il-FABP), brain-type (FABP7/B-FABP), myelin-type (FABP8/M-FABP), and testis-type FABPs (FABP9/T-FABP) [1]. However, the tissue/cell-type classification of FABPs is somewhat misleading, since no FABP is exclusively specific to a given tissue or cell type, and most tissues express several FABP isoforms [1]: e.g., FABP1 in the kidney and intestine, FABP2 in the liver, FABP3 in liver, FABP4 in macrophages, and FABP5 in adipocytes, macrophages, liver, and heart.

Numerous studies have recently shown the presence of FABPs in circulation. Since FABPs lack a secretory signal sequence, the presence of FABPs in serum has been considered to be a promising tissue-specific marker of tissue injury: FABP1 for liver damage [3], FABP2 for intestinal injury [4], [5], and FABP3 for acute myocardial infarction and ongoing myocardial damage in heart failure [6], [7]. However, it has recently been reported that FABP4 is secreted from adipocytes [8]. Furthermore, increased serum concentration of FABP4 has been shown to be associated with obesity, type 2 diabetes, hypertension, and cardiovascular diseases [8]–[11]. Similar findings have also been reported for FABP5 [12], [13]. However, the significance of serum concentrations of FABPs in the general population has not been elucidated. In the present study, we determined serum concentrations of FABP1, FABP2, FABP3, FABP4, and FABP5 in Japanese subjects on no medication and investigated the relationships of the concentration of each FABP isoform with tissue damage and metabolic phenotype.

## Methods

### Study population

In the Tanno-Sobetsu Study, a study with a population-based cohort design, a total of 617 Japanese subjects (male/female: 260/357, mean age: 65.8±0.5 years) were recruited from residents of two rural towns, Tanno and Sobetsu, in Hokkaido, the northernmost island of Japan, in 2011. Subjects who were being treated with any medications were excluded, and subjects who were not on any medication (n = 296, male/female: 122/174) were enrolled in the present analyses. This study conformed to the principles outlined in the Declaration of Helsinki and was performed with the approval of the institutional ethical committee of Sapporo Medical University. Written informed consent was received from all of the subjects.

### Measurements

Medical check-ups were performed between 06:00 h and 09:00 h after an overnight fast. After measuring anthropometric parameters, blood pressure was measured twice consecutively on the upper arm using an automated sphygmomanometer (HEM-907, Omron Co., Kyoto, Japan) with subjects in a seated resting position, and average blood pressure was used for analysis. Body mass index (BMI) was calculated as body weight (in kilograms) divided by the square of body height (in meters).

Peripheral venous blood samples were obtained from study subjects after physical examination for complete blood count and biochemical analyses of the serum. The serum samples were analyzed immediately or stored at −80°C until biochemical analyses. Concentrations of FABPs in serum samples were measured using commercially available enzyme-linked immunosorbent assay kits for FABP1 (CIMIC Co., Tokyo, Japan), FABP2 (Hycult Biotech, Uden, Netherlands), FABP3 (DS Pharma Biomedical Co., Osaka, Japan), FABP4 (Biovendor R&D, Modrice, Czech Republic), and FABP5 (USCN Life Science, Houston, U.S.A.). The accuracy, precision and reproducibility of the kits for FABP1, FABP2, FABP3, and FABP4 have been described previously [8], [14]–[16]. The intra- and inter-assay coefficient variances in the kits were <15%. According to manufacturer's protocol, no cross-reactivity of FABP5 with other FABP types was observed. Fasting plasma glucose was determined by the glucose oxidase method. Fasting plasma insulin was measured by a radioimmunoassay method (Insulin RIA bead, Dianabot, Tokyo, Japan). Creatinine (Cr), aspartate transaminase (AST), alanine aminotransferase (ALT), and lipid profiles, including total cholesterol, high-density lipoprotein (HDL) cholesterol, and triglycerides, were determined by enzymatic methods. Low-density lipoprotein (LDL) cholesterol level was calculated by the Friedewald equation. Brain natriuretic peptide (BNP) was measured using an assay kit (Shionogi & Co., Osaka, Japan). High-sensitivity C-reactive protein (hsCRP) was measured by a nephelometry method.

As an index of renal function, estimated GFR (eGFR) was calculated by an equation for Japanese [17]: eGFR (mL/min/1.73 m^2^) = 194×Cr^(−1.094)^×age^(−0.287)^×0.739 (if female). HOMA-R, an indicator of insulin resistance, was calculated by the previously reported formula: insulin (μU/ml)×glucose (mg/dl)/405.

### Statistical analysis

Numeric variables are expressed as means ± SEM. The distribution of each parameter was tested for its normality using the Shapiro-Wilk W test, and non-normally distributed parameters were logarithmically transformed. Comparison between two groups was done with an unpaired *t* test. The correlation between two variables was evaluated using Pearson's correlation coefficient. Multiple linear regression analysis was performed to identify independent determinants of each FABP concentration and HOMA-R using the variables with a significant and non-confounding correlation in simple regression analysis as independent predictors, showing the t-ratio calculated as the ratio of regression coefficient and standard error of regression coefficient and the percentage of variance in the each FABP concentration or HOMA-R that they explained (R^2^). A p value of less than 0.05 was considered statistically significant. All data were analyzed by using JMP 9 for Macintosh (SAS Institute, Cary, NC).

## Results

### Serum levels of FABPs

**Figure 1:**
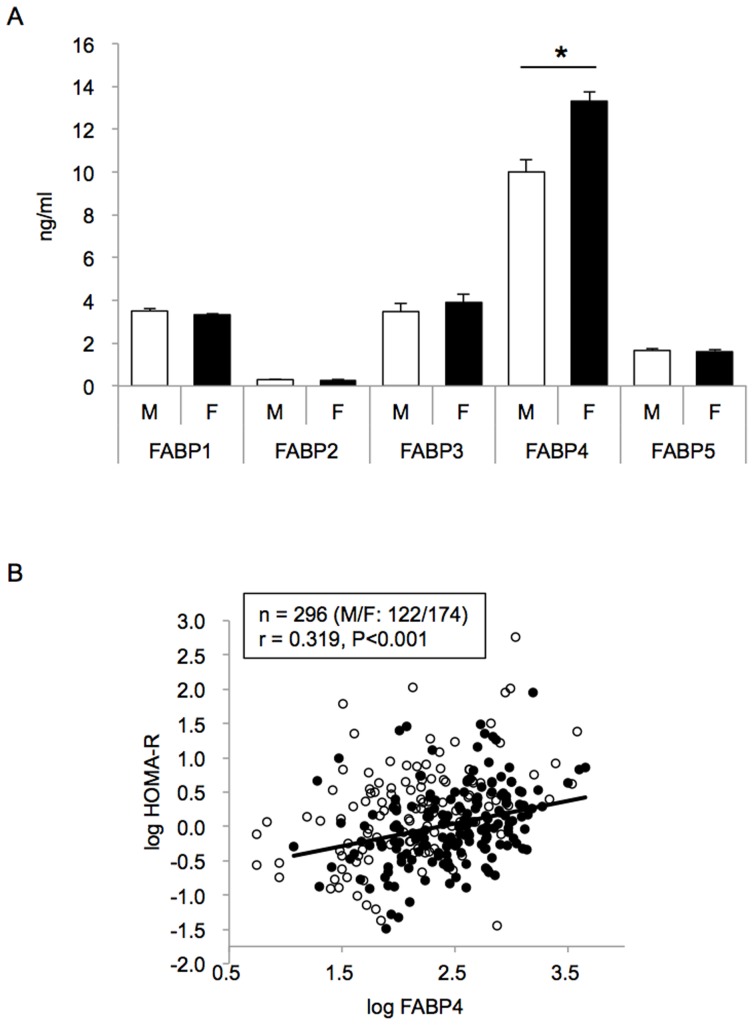
Concentrations of FABPs. A. Bar graphs show serum concentrations of FABP1, FABP2, FABP3, FABP4, and FABP5 in male (n = 122) and female (n = 174) subjects on no medication. Values are presented as means ± SEM. *P<0.001. B. Log HOMA-R was plotted against log FABP4 in each study subject. There was a significant correlation between the two parameters (n = 296, r = 0.319, P<0.001). Open circle: male (n = 122), closed circle: female (n = 174).

Demographic characteristics of the 296 recruited subjects (male/female: 122/174) are shown in Table 1. There was no significant difference in age, systolic blood pressure, eGFR, and BNP level between the male and female subjects. Total, HDL, and LDL cholesterol levels were significantly higher in females than in males. Indices of adiposity (BMI and waist circumference), indices of glucose metabolism (glucose, insulin, and HOMA-R), and Cr, AST, ALT, and hsCRP were higher in males than in females. There were approximately 30-fold differences in serum levels of FABPs depending on the isoform: FABP1 (male, female: 3.4±0.1, 3.3±0.1 ng/ml), FABP2 (male, female: 0.30±0.01, 0.27±0.01 ng/ml), FABP3 (male, female: 3.5±0.4, 3.9±0.4 ng/ml), FABP4 (male, female: 10.0±0.6, 13.3±0.5 ng/ml), and FABP5 (male, female: 1.8±0.1, 1.7±0.1 ng/ml) (Figure 1A). FABP4 concentration was the highest among levels of FABPs and was significantly higher in females than in males. No other concentrations of FABPs showed a significant gender difference.

**Table 1:** Characteristics of the studied subjects.

	Total	Male	Female
n	296	122	174
Age (years)	60.4±0.9	60.8±1.4	60.1±1.1
Body mass index (kg/m^2^)	22.7±0.2	23.5±0.3	22.1±0.2**
Waist circumference (cm)	82.9±0.6	85.8±0.9	80.8±0.7**
Systolic blood pressure (mmHg)	133.2±1.4	135.1±2.0	131.8±1.9
Diastolic blood pressure (mmHg)	76.5±0.7	78.4±1.2	75.2±0.9*
Biochemical data			
Total cholesterol (mg/dl)	203.3±2.0	195.0±3.1	209.0±2.5**
HDL cholesterol (mg/dl)	67.9±1.1	59.5±1.6	73.8±1.3**
LDL cholesterol (mg/dl)	121.9±1.7	117.8±2.8	124.7±2.2*
Triglycerides (mg/dl)	109.0±6.1	115.9±5.7	84.9±2.9**
Glucose (mg/dl)	97.7±3.0	97.9±1.6	92.3±1.0**
HbA1c (%)	5.03±0.03	5.11±0.04	4.97±0.03*
Insulin (μU/ml)	5.8±0.3	6.7±0.5	5.2±0.2**
HOMA-R	1.41±0.08	1.69±0.17	1.22±0.07**
Cr (mg/dl)	0.74±0.01	0.84±0.01	0.66±0.01**
estimated GFR (ml/min/1.73 m^2^)	73.8±0.8	75.2±1.3	72.8±1.1
AST (IU/l)	23.6±0.5	26.1±0.9	21.8±0.5**
ALT (IU/l)	21.5±0.7	26.3±1.3	18.1±0.7**
BNP (pg/ml)	26.2±1.9	24.1±3.7	27.7±1.9
hsCRP (mg/dl)	0.064±0.005	0.083±0.009	0.052±0.005**

Variables are expressed as n or means ± SEM.

* P<0.05,

** P<0.01 vs. male.

### Correlations between FABPs levels and clinical parameters

Table 2 shows a summary of the results of univariate regression analyses for each FABP isoform. Serum levels of all of the FABPs were negatively correlated with eGFR. FABP1 level was positively correlated with age, systolic blood pressure, triglycerides, AST, ALT, and BNP. None of the parameters determined in this study, except for eGFR, was significantly correlated with FABP2 concentration, although there were trends for FABP2 to correlate with triglycerides (r = 0.11, p = 0.058) and to negatively correlate with HDL cholesterol (r = −0.11, p = 0.050). FABP3 level was positively correlated with age, systolic and diastolic blood pressures, and BNP. FABP4 level was negatively correlated with HDL cholesterol and was positively correlated with age, BMI, waist circumference, systolic and diastolic blood pressures, total and LDL cholesterol, triglycerides, glucose, insulin, HOMA-R, BNP, and hsCRP. FABP5 level correlated positively with age, waist circumference, systolic blood pressure, glucose, HOMA-R, AST, ALT, and BNP.

**Table 2:** Simple regression analysis for log FABPs.

	log FABP1		log FABP2		log FABP3		log FABP4		log FABP5	
	r	p	r	p	r	p	r	p	r	p
Age	0.201	0.001*	0.073	0.210	0.232	<0.001*	0.309	<0.001*	0.348	<0.001*
Body mass index	0.107	0.068	−0.009	0.872	−0.002	0.975	0.454	<0.001*	0.110	0.059
Waist circumference	0.103	0.078	−0.004	0.949	0.015	0.804	0.458	<0.001*	0.141	0.015*
Systolic blood pressure	0.154	0.008*	0.022	0.712	0.187	0.001*	0.257	<0.001*	0.193	<0.001*
Diastolic blood pressure	0.046	0.430	−0.001	0.998	0.137	0.018*	0.214	<0.001*	0.067	0.251
Total cholesterol	0.092	0.118	0.026	0.661	−0.003	0.957	0.255	<0.001*	0.037	0.532
LDL cholesterol	0.077	0.186	0.066	0.262	0.059	0.315	0.257	<0.001*	0.013	0.823
HDL cholesterol	0.011	0.848	−0.114	0.050	0.043	0.461	−0.075	0.196	−0.042	0.467
log Triglycerides	0.125	0.032*	0.110	0.058	−0.112	0.054	0.187	0.001*	0.085	0.145
Glucose	0.074	0.208	0.033	0.578	−0.008	0.889	0.154	0.008*	0.175	0.003*
log Insulin	−0.011	0.855	−0.002	0.977	0.031	0.600	0.311	<0.001*	0.112	0.054
log HOMA-R	0.006	0.924	0.005	0.927	0.034	0.562	0.319	<0.001*	0.143	0.014*
eGFR	−0.119	0.042*	−0.160	0.006*	−0.229	<0.001*	−0.386	<0.001*	−0.331	<0.001*
log AST	0.232	<0.001*	0.088	0.132	0.104	0.075	0.112	0.055	0.186	0.001*
log ALT	0.226	<0.001*	0.059	0.313	0.061	0.299	0.016	0.781	0.134	0.022*
log BNP	0.156	0.007*	0.065	0.269	0.211	<0.001*	0.176	0.002*	0.175	0.003*
log hsCRP	0.067	0.260	−0.021	0.722	0.083	0.163	0.183	0.002*	0.108	0.068

Results of multiple regression analyses for each FABP isoform are shown in Table 3. As independent determinants, ALT for FABP1, eGFR for FABP2 and FABP3, age, gender, waist circumference, and eGFR for FABP4, and age and eGFR for FABP5 were selected (Table 3).

**Table 3:** Multiple regression analysis for log FABPs.

	FABP1		FABP2		FABP3		FABP4		FABP5	
	t	p	t	p	t	p	t	p	t	p
Age	1.27	0.205	−0.37	0.711	0.51	0.609	2.11	0.035*	3.02	0.003*
Gender (Male)	0.01	0.995	1.41	0.159	0.87	0.383	−8.69	<0.001*	0.09	0.930
Waist circumference	-	-	-	-	-	-	8.59	<0.001*	0.37	0.711
Systolic blood pressure	0.38	0.706	-	-	1.14	0.256	−0.09	0.931	−0.53	0.598
Total cholesterol	-	-	-	-	-	-	1.76	0.08	-	-
log Triglycerides	0.64	0.522	-	-	-	-	-	-	-	-
log HOMA-R	-	-	-	-	-	-	1.33	0.185	1.23	0.220
eGFR	−0.4	0.687	−2.63	0.009*	−2.12	0.035*	−5.07	<0.001*	−3.16	0.002*
log ALT	3.54	0.001*	-	-	-	-	-	-	1.90	0.059
log BNP	1.35	0.178	-	-	1.8	0.073	−1.4	0.162	−0.01	0.996
log hsCRP	-	-	-	-	-	-	0.56	0.573	-	-
	R^2^ = 0.10		R^2^ = 0.03		R^2^ = 0.09		R^2^ = 0.51		R^2^ = 0.18	

### FABP4 level as a metabolic biomarker in the general population

Results of univariate and multivariate regression analyses (Tables 2 and 3) indicated that FABP4 is the most strongly related to metabolic markers among FABPs. Hence, we next focused on the significance of FABP4 in metabolic disorders, especially insulin resistance, in the study subjects. Serum FABP4 level was positively correlated with HOMA-R (r = 0.32, p<0.001) as shown in Figure 1B. When the subjects were divided into male and female subjects, there was a still significant correlation between FABP4 level and HOMA-R in each gender (male: r = 0.40, p<0.001; female: r = 0.38, p<0.001) (Table 4). HOMA-R was also negatively correlated with HDL cholesterol and was positively correlated with age, BMI, waist circumference, systolic and diastolic blood pressures, LDL cholesterol, triglycerides, and hsCRP (Table 4). Multiple regression analysis using age, gender, and the non-confounding correlated parameters revealed that serum FABP4 concentration was an independent predictor of HOMA-R, explaining a total of 40.6% of the variance in this measure (R^2^ = 0.406) (Table 5).

**Table 4:** Simple regression analysis for log HOMA-R.

	Total		Male		Female	
	r	p	r	p	r	p
Age	0.030	0.609	−0.030	0.744	0.080	0.292
Body mass index	0.558	<0.001*	0.602	<0.001*	0.491	<0.001*
Waist circumference	0.545	<0.001*	0.569	<0.001*	0.500	<0.001*
Systolic blood pressure	0.234	<0.001*	0.160	0.079	0.287	<0.001*
Diastolic blood pressure	0.215	<0.001*	0.121	0.185	0.276	<0.001*
Total cholesterol	0.018	0.760	0.159	0.081	−0.048	0.534
LDL cholesterol	0.127	0.029*	0.224	0.014*	0.076	0.319
HDL cholesterol	−0.394	<0.001*	−0.389	<0.001*	−0.348	<0.001*
log Triglycerides	0.376	<0.001*	0.410	<0.001*	0.289	<0.001*
log hsCRP	0.227	<0.001*	0.236	0.010*	0.177	0.022*
log FABP4	0.319	<0.001*	0.403	<0.001*	0.378	<0.001*

**Table 5:** Multiple regression analysis for log HOMA-R.

	t	p
Age	−3.32	0.001*
Gender (Male)	−0.23	0.821
Waist circumference	6.49	<0.001*
Systolic blood pressure	3.3	0.001*
HDL cholesterol	−4.42	<0.001*
log hsCRP	0.62	0.539
log FABP4	2.03	0.044*
	R^2^ = 0.41	

## Discussion

To the best of our knowledge, this is the first report on the circulating level of each FABP in a general population on no medications. Although none of the FABPs are tissue-specific, the FABP family has drawn interest recently as early and sensitive serum markers of tissue damage or injury. The present study showed that concentrations of FABPs except for FABP2 were correlated with distinct biochemical markers reflecting tissue damage in subjects on no medication: i.e., FABP1 for liver damage, FABP3 for cardiac injury, FABP4 for adiposity and metabolic syndrome, and FABP5 for adiposity, insulin resistance, cardiac injury, and liver damage. FABP2 was not correlated with any tissue injury markers, presumably because none of the clinical parameters determined in this study are specific and sensitive markers of intestinal injury. In addition, we found that serum concentrations of FABP1∼FABP5 negatively correlated with eGFR. The results are consistent with results of previous studies showing that levels of several FABPs were increased in subjects with renal dysfunction [3], [18]–[20] and indicate that FABPs are eliminated from the circulation mainly by renal clearance. Hence, though eGFR needs to be taken into account in interpretation of serum FABP levels, relatively high tissue concentrations of FABPs and their low serum concentrations under normal conditions would enable serum FABP levels to be novel and sensitive biomarkers.

### FABP1

No influence of gender was observed for FABP1 in serum, being consistent with a previous report [3]. FABP1 level was correlated with AST and ALT (Table 2), potentially reflecting liver injury. Serum AST, ALT and lactate dehydrogenease are commonly used as plasma markers of acute hepatocellular injury for detection and monitoring of liver disease. Although ALT is a well-established specific, quickly measurable and cost-effective biomarker of hepatocellular injury, it is a relatively large protein (96 kDa) and slowly indicates cell damage. A study recruiting liver transplant recipients showed that elevation of serum FABP1 level after hepatocellular injury due to rejection was larger and faster than that of ALT, indicating that FABP1 is a more sensitive marker [3]. In the present study, we recruited only apparently healthy subjects on no medication and confirmed that their serum biochemical parameters, including AST and ALT, were within normal ranges. Thus, correlation between serum FABP1 concentration and “normal” levels of AST and ALT indicates that serum FABP1 is very sensitive marker of liver injury or injurious stress on the liver.

Regarding metabolic phenotype, it has been reported that FABP1-deficient mice were of normal weight and that serum levels of triglycerides and fatty acids were unchanged [21], [22]. Changes in metabolic parameters caused by a high fat/cholesterol diet in FABP1-deficient mice differed between studies [23], [24]. It is possible that the principal action of FABP1 is not serum lipid regulation but another action. Interestingly, recent studies have shown that urinary FABP1 in humans would be a useful clinical marker for prediction and monitoring of the progression of renal diseases [14], [25]. In the present study, serum FABP1 concentration was correlated with systolic blood pressure and BNP, indicating a possible role in cardiovascular regulation. Since subjects with heart failure were generally excluded from the study subjects, it is unlikely that correlation of FABP1 and BNP is attributable to liver congestion by latent right ventricular failure.

### FABP2

A polymorphism in FABP2, an alanine-to-threonine substitution at codon 54 (Thr-54), has been reported to be associated with insulin resistance in Pima Indians, a population with an extremely high prevalence of obesity and type 2 diabetes [26]. However, the association between the Thr-54 allele and insulin resistance in other populations has been controversial [27], [28]. Previous studies showed the applicability of FABP2 for detection of intestinal injury after acute ischemic diseases, rejection and necrotic enterocolitis [4], [5], whereas FABP2 concentrations in plasma of healthy individuals were reported to be undetectable or very low [5], [29]. In the present study, we could detect FABP2 in serum of healthy subjects, and its level was lowest among FABPs. In contrast to the other FABP isoforms determined in this study, FABP2 level was not correlated with parameters relevant to glucose and lipid metabolism, blood pressure or BNP. These features of FABP2 appear to be advantageous as a biomarker of intestinal injury.

### FABP3

FABP3 is abundant in the myocardium and is rapidly released from cells into the circulation after onset of cardiomyocyte damage. Serum concentration of FABP3 has been characterized as an early biochemical marker of acute myocardial infarction and a sensitive marker of myocardial damage in patients with heart failure [6], [7]. In the present study, serum FABP3 level positively correlated with blood pressure, BNP and eGFR (Table 2), although only eGFR was selected as a significant determinant by multivariate analysis (Table 3). These results suggest that the association of FABP3 with blood pressure and BNP was mediated by decline of eGFR. However, recent studies have shown diastolic ventricular dysfunction in approximately 20% of asymptomatic subjects in the general population. Thus, it is possible that elevation of FABP3 after adjustment with eGFR indicates such asymptomatic ventricular dysfunction.

There was no significant gender difference in FABP3 level in subjects on no medication (Figure 1A). In contrast, higher serum FABP3 levels in males than in females were reported earlier in Japanese and Caucasian subjects, and the gender difference was attributed to larger muscle mass in males [30]–[32]. However, the study using Japanese subjects in the earlier studies [30], [31] included those with hypertension, diabetes mellitus, dyslipidemia, and smoking habits, and incidences of coronary risk factors were higher in males than in females [30], [31]. Thus, the reported gender difference in serum FABP3 level in Japanese subjects is likely to be an apparent one due to different myocardial insults between male and female groups. On the other hand, the other earlier study enrolled Caucasian healthy subjects and young volunteers [32]. It is also possible that there is a difference in muscle tissue distribution between Japanese and Caucasian subjects.

### FABP4

Previous studies using animal models indicated that FABP4 plays a significant role in several aspects of metabolic syndrome, including insulin resistance, type 2 diabetes, and atherosclerosis, through its action at the interface of metabolic and inflammatory pathways in adipocytes and macrophages [33]–[36]. It has also been demonstrated that chemical inhibition of FABP4 could be a therapeutic strategy against insulin resistance, diabetes mellitus, fatty liver disease, and atherosclerosis in experimental models [37]. Interestingly, it has recently been shown that FABP4 is secreted from adipocytes, though there is no typical sequence of secretory signal peptides [8]. We previously confirmed that FABP4 release from adipocytes was not an escape due to apoptosis or necrosis of adipocytes [36], although the precise mechanisms underlying secretion of FABPs are still unclear. In this study, serum concentration of FABP4 was higher in females than in males, being consistent with results of previous studies [8]–[11], [20]. This may be due to the larger amount of body fat in females than in males. Recent clinical studies have shown that elevation of serum FABP4 is associated with obesity, insulin resistance, hypertension, and atherosclerosis [8]–[11]. We and others have also shown that serum FABP4 level predicts long-term cardiovascular outcomes [20], [38].

In the present study, we found that FABP4 level is associated with clinical parameters of obesity, insulin resistance, dyslipidemia, and high blood pressure even in asymptomatic apparently healthy subjects with no pharmacological treatments. These findings raise the possibility that elevation of serum FABP4 is a very early event in the pathogenesis of insulin resistance and obesity. However, it is still unknown whether association of elevated circulating FABP4 level with insulin resistance is a result of direct physiological effects of FABP4 as a bioactive molecule *in vivo*. To address this issue, effects of recombinant FABP on metabolic phenotype need to be clearly demonstrated.

### FABP5

FABP5 is expressed most abundantly in epidermal cells of the skin and is also present in other tissues, including adipocytes, macrophages, liver, and heart [1]. Ablation of FABP5 led to a mild increase in systemic insulin sensitivity in genetic and dietary obesity mouse models [39], whereas adipose tissue-specific overexpression of FABP5 in transgenic mice resulted in a reduction in systemic insulin sensitivity in a high-fat diet model [39]. In the present study, serum level of FABP5 was correlated with waist circumference and HOMA-R, indicating that FABP5 would be a metabolic marker, although the correlation was not as strong as that of FABP4.

In addition to metabolic parameters, AST and ALT levels were correlated with FABP5. Several lines of evidence indicate a role of FABP5 in the liver. Complete or partial lack of FABP5 in the liver during perinatal development was compensated by overexpression of FABP3 [40]. The expression of FABP5, but not that of FABP1, was increased in liver parenchymal cells by a western-type high-cholesterol diet in atherosclerotic LDL receptor-deficient mice [41]. These findings suggest that FABP5 has important and distinct roles in the liver, although there were no apparent morphological changes in the liver of FABP5-deficient mice [40]. Whether elevation of serum FABP5 and that of FABP1 indicate different types of liver injury is an interesting issue and remains to be investigated.

### Study limitations

Our study has some limitations. First, this study is a cross-sectional design, which does not prove causal relations between serum levels of FABPs and the correlated biomarkers. A longitudinal study and interventional study are needed to clarify what underlies the relationship between FABPs and metabolic and tissue damage markers. Second, because the subjects of our study were only Japanese people, it is unclear whether the present findings can be generalized to other ethnicities. Lastly, it is not clear whether methods of each FABP isoform assay used in this study were suitable for diagnostic and prognostic use in terms of timely and definitive evaluation in clinical practice.

## Conclusions

Serum levels of FAPB1∼FABP5 showed distinct patterns of correlation with physiological and metabolic parameters in a general population without pharmacological treatments, although eGFR is a negative determinant of all FABP isoform levels. Of serum FABPs, FABP4 showed the closest correlations with metabolic parameters and was the only independent determinant of HOMA-R in a general population. Thus, this FABP isoform might be an early and useful biomarker of metabolic syndrome phenotype.

## References

[1] Furuhashi M, Hotamisligil GS (2008) Fatty acid-binding proteins: role in metabolic diseases and potential as drug targets. Nat Rev Drug Discov 7: 489–503. 18511927 10.1038/nrd2589 PMC2821027

[2] Furuhashi M, Ishimura S, Ota H, Miura T (2011) Lipid chaperones and metabolic inflammation. Int J Inflam 2011: 642612. 22121495 10.4061/2011/642612 PMC3206330

[3] Pelsers MM, Morovat A, Alexander GJ, Hermens WT, Trull AK, et al (2002) Liver fatty acid-binding protein as a sensitive serum marker of acute hepatocellular damage in liver transplant recipients. Clin Chem 48: 2055–2057. 12406996

[4] Pelsers MM, Hermens WT, Glatz JF (2005) Fatty acid-binding proteins as plasma markers of tissue injury. Clin Chim Acta 352: 15–35. 15653098 10.1016/j.cccn.2004.09.001

[5] Pelsers MM, Namiot Z, Kisielewski W, Namiot A, Januszkiewicz M, et al (2003) Intestinal-type and liver-type fatty acid-binding protein in the intestine. Tissue distribution and clinical utility. Clin Biochem 36: 529–535. 14563446 10.1016/s0009-9120(03)00096-1

[6] Tanaka T, Hirota Y, Sohmiya K, Nishimura S, Kawamura K (1991) Serum and urinary human heart fatty acid-binding protein in acute myocardial infarction. Clin Biochem 24: 195–201. 2040092 10.1016/0009-9120(91)90571-u

[7] Setsuta K, Seino Y, Ogawa T, Arao M, Miyatake Y, et al (2002) Use of cytosolic and myofibril markers in the detection of ongoing myocardial damage in patients with chronic heart failure. Am J Med 113: 717–722. 12517360 10.1016/s0002-9343(02)01394-3

[8] Xu A, Wang Y, Xu JY, Stejskal D, Tam S, et al (2006) Adipocyte fatty acid-binding protein is a plasma biomarker closely associated with obesity and metabolic syndrome. Clin Chem 52: 405–413. 16423904 10.1373/clinchem.2005.062463

[9] Tso AW, Xu A, Sham PC, Wat NM, Wang Y, et al (2007) Serum adipocyte fatty acid binding protein as a new biomarker predicting the development of type 2 diabetes: a 10-year prospective study in a Chinese cohort. Diabetes Care 30: 2667–2672. 17620449 10.2337/dc07-0413

[10] Yeung DC, Xu A, Cheung CW, Wat NM, Yau MH, et al (2007) Serum adipocyte fatty acid-binding protein levels were independently associated with carotid atherosclerosis. Arterioscler Thromb Vasc Biol 27: 1796–1802. 17510463 10.1161/ATVBAHA.107.146274

[11] Ota H, Furuhashi M, Ishimura S, Koyama M, Okazaki Y, et al (2012) Elevation of fatty acid-binding protein 4 is predisposed by family history of hypertension and contributes to blood pressure elevation. Am J Hypertens 25: 1124–1130. 22717543 10.1038/ajh.2012.88 PMC3449332

[12] Yeung DC, Wang Y, Xu A, Cheung SC, Wat NM, et al (2008) Epidermal fatty-acid-binding protein: a new circulating biomarker associated with cardio-metabolic risk factors and carotid atherosclerosis. Eur Heart J 29: 2156–2163. 18603624 10.1093/eurheartj/ehn295

[13] Bagheri R, Qasim AN, Mehta NN, Terembula K, Kapoor S, et al (2010) Relation of plasma fatty acid binding proteins 4 and 5 with the metabolic syndrome, inflammation and coronary calcium in patients with type-2 diabetes mellitus. Am J Cardiol 106: 1118–1123. 20920650 10.1016/j.amjcard.2010.06.028 PMC3108486

[14] Kamijo A, Kimura K, Sugaya T, Yamanouchi M, Hikawa A, et al (2004) Urinary fatty acid-binding protein as a new clinical marker of the progression of chronic renal disease. J Lab Clin Med 143: 23–30. 14749682 10.1016/j.lab.2003.08.001

[15] Morariu AM, Loef BG, Aarts LP, Rietman GW, Rakhorst G, et al (2005) Dexamethasone: benefit and prejudice for patients undergoing on-pump coronary artery bypass grafting: a study on myocardial, pulmonary, renal, intestinal, and hepatic injury. Chest 128: 2677–2687. 16236942 10.1378/chest.128.4.2677

[16] Ohkaru Y, Asayama K, Ishii H, Nishimura S, Sunahara N, et al (1995) Development of a sandwich enzyme-linked immunosorbent assay for the determination of human heart type fatty acid-binding protein in plasma and urine by using two different monoclonal antibodies specific for human heart fatty acid-binding protein. J Immunol Methods 178: 99–111. 7829870 10.1016/0022-1759(94)00248-u

[17] Matsuo S, Imai E, Horio M, Yasuda Y, Tomita K, et al (2009) Revised equations for estimated GFR from serum creatinine in Japan. Am J Kidney Dis 53: 982–992. 19339088 10.1053/j.ajkd.2008.12.034

[18] Furuhashi M, Ura N, Hasegawa K, Yoshida H, Tsuchihashi K, et al (2003) Serum ratio of heart-type fatty acid-binding protein to myoglobin. A novel marker of cardiac damage and volume overload in hemodialysis patients. Nephron Clin Pract 93: C69–74. 12616033 10.1159/000068520

[19] Furuhashi M, Ura N, Hasegawa K, Tsuchihashi K, Nakata T, et al (2004) Utility of serum ratio of heart-type fatty acid-binding protein to myoglobin for cardiac damage regardless of renal dysfunction. Circ J 68: 656–659. 15226631 10.1253/circj.68.656

[20] Furuhashi M, Ishimura S, Ota H, Hayashi M, Nishitani T, et al (2011) Serum fatty acid-binding protein 4 is a predictor of cardiovascular events in end-stage renal disease. PLoS ONE 6: e27356. 22102888 10.1371/journal.pone.0027356 PMC3213139

[21] Martin GG, Danneberg H, Kumar LS, Atshaves BP, Erol E, et al (2003) Decreased liver fatty acid binding capacity and altered liver lipid distribution in mice lacking the liver fatty acid-binding protein gene. J Biol Chem 278: 21429–21438. 12670956 10.1074/jbc.M300287200

[22] Newberry EP, Xie Y, Kennedy S, Han X, Buhman KK, et al (2003) Decreased hepatic triglyceride accumulation and altered fatty acid uptake in mice with deletion of the liver fatty acid-binding protein gene. J Biol Chem 278: 51664–51672. 14534295 10.1074/jbc.M309377200

[23] Martin GG, Atshaves BP, McIntosh AL, Mackie JT, Kier AB, et al (2006) Liver fatty acid binding protein gene ablation potentiates hepatic cholesterol accumulation in cholesterol-fed female mice. Am J Physiol Gastrointest Liver Physiol 290: G36–48. 16123197 10.1152/ajpgi.00510.2004

[24] Newberry EP, Xie Y, Kennedy SM, Luo J, Davidson NO (2006) Protection against Western diet-induced obesity and hepatic steatosis in liver fatty acid-binding protein knockout mice. Hepatology 44: 1191–1205. 17058218 10.1002/hep.21369

[25] Kamijo-Ikemori A, Sugaya T, Kimura K (2006) Urinary fatty acid binding protein in renal disease. Clin Chim Acta 374: 1–7. 16860300 10.1016/j.cca.2006.05.038

[26] Baier LJ, Sacchettini JC, Knowler WC, Eads J, Paolisso G, et al (1995) An amino acid substitution in the human intestinal fatty acid binding protein is associated with increased fatty acid binding, increased fat oxidation, and insulin resistance. J Clin Invest 95: 1281–1287. 7883976 10.1172/JCI117778 PMC441467

[27] Stan S, Lambert M, Delvin E, Paradis G, O'Loughlin J, et al (2005) Intestinal fatty acid binding protein and microsomal triglyceride transfer protein polymorphisms in French-Canadian youth. J Lipid Res 46: 320–327. 15547295 10.1194/jlr.M400346-JLR200

[28] Ito K, Nakatani K, Fujii M, Katsuki A, Tsuchihashi K, et al (1999) Codon 54 polymorphism of the fatty acid binding protein gene and insulin resistance in the Japanese population. Diabet Med 16: 119–124. 10229304 10.1046/j.1464-5491.1999.00034.x

[29] Guthmann F, Borchers T, Wolfrum C, Wustrack T, Bartholomaus S, et al (2002) Plasma concentration of intestinal- and liver-FABP in neonates suffering from necrotizing enterocolitis and in healthy preterm neonates. Mol Cell Biochem 239: 227–234. 12479590

[30] Niizeki T, Takeishi Y, Takabatake N, Shibata Y, Konta T, et al (2007) Circulating levels of heart-type fatty acid-binding protein in a general Japanese population: effects of age, gender, and physiologic characteristics. Circ J 71: 1452–1457. 17721027 10.1253/circj.71.1452

[31] Narumi T, Shishido T, Kiribayashi N, Kadowaki S, Nishiyama S, et al (2012) Impact of insulin resistance on silent and ongoing myocardial damage in normal subjects: the Takahata study. Exp Diabetes Res 2012: 815098. 23093954 10.1155/2012/815098 PMC3474255

[32] Pelsers MM, Chapelle JP, Knapen M, Vermeer C, Muijtjens AM, et al (1999) Influence of age and sex and day-to-day and within-day biological variation on plasma concentrations of fatty acid-binding protein and myoglobin in healthy subjects. Clin Chem 45: 441–443. 10053065

[33] Hotamisligil GS, Johnson RS, Distel RJ, Ellis R, Papaioannou VE, et al (1996) Uncoupling of obesity from insulin resistance through a targeted mutation in aP2, the adipocyte fatty acid binding protein. Science 274: 1377–1379. 8910278 10.1126/science.274.5291.1377

[34] Makowski L, Boord JB, Maeda K, Babaev VR, Uysal KT, et al (2001) Lack of macrophage fatty-acid-binding protein aP2 protects mice deficient in apolipoprotein E against atherosclerosis. Nat Med 7: 699–705. 11385507 10.1038/89076 PMC4027052

[35] Maeda K, Cao H, Kono K, Gorgun CZ, Furuhashi M, et al (2005) Adipocyte/macrophage fatty acid binding proteins control integrated metabolic responses in obesity and diabetes. Cell Metab 1: 107–119. 16054052 10.1016/j.cmet.2004.12.008

[36] Furuhashi M, Fucho R, Gorgun CZ, Tuncman G, Cao H, et al (2008) Adipocyte/macrophage fatty acid-binding proteins contribute to metabolic deterioration through actions in both macrophages and adipocytes in mice. J Clin Invest 118: 2640–2650. 18551191 10.1172/JCI34750 PMC2423863

[37] Furuhashi M, Tuncman G, Gorgun CZ, Makowski L, Atsumi G, et al (2007) Treatment of diabetes and atherosclerosis by inhibiting fatty-acid-binding protein aP2. Nature 447: 959–965. 17554340 10.1038/nature05844 PMC4076119

[38] von Eynatten M, Breitling LP, Roos M, Baumann M, Rothenbacher D, et al (2012) Circulating adipocyte fatty acid-binding protein levels and cardiovascular morbidity and mortality in patients with coronary heart disease: a 10-year prospective study. Arterioscler Thromb Vasc Biol 32: 2327–2335. 22679309 10.1161/ATVBAHA.112.248609

[39] Maeda K, Uysal KT, Makowski L, Gorgun CZ, Atsumi G, et al (2003) Role of the fatty acid binding protein mal1 in obesity and insulin resistance. Diabetes 52: 300–307. 12540600 10.2337/diabetes.52.2.300 PMC4027060

[40] Owada Y, Suzuki I, Noda T, Kondo H (2002) Analysis on the phenotype of E-FABP-gene knockout mice. Mol Cell Biochem 239: 83–86. 12479572

[41] Hoekstra M, Stitzinger M, van Wanrooij EJ, Michon IN, Kruijt JK, et al (2006) Microarray analysis indicates an important role for FABP5 and putative novel FABPs on a Western-type diet. J Lipid Res 47: 2198–2207. 16885566 10.1194/jlr.M600095-JLR200

